# Keratin 13 Is Enriched in Prostate Tubule-Initiating Cells and May Identify Primary Prostate Tumors that Metastasize to the Bone

**DOI:** 10.1371/journal.pone.0163232

**Published:** 2016-10-06

**Authors:** Sandy Liu, Radu M. Cadaneanu, Baohui Zhang, Lihong Huo, Kevin Lai, Xinmin Li, Colette Galet, Tristan R. Grogan, David Elashoff, Stephen J. Freedland, Matthew Rettig, William J. Aronson, Beatrice S. Knudsen, Michael S. Lewis, Isla P. Garraway

**Affiliations:** 1 Department of Hematology-Oncology, David Geffen School of Medicine at UCLA, Los Angeles, California, United States of America; 2 Department of Urology, David Geffen School of Medicine at UCLA, Los Angeles, California, United States of America; 3 Department of Pathology and Laboratory Medicine, David Geffen School of Medicine at UCLA, Los Angeles, California, United States of America; 4 Department of Medicine Statistics Core, David Geffen School of Medicine at UCLA, Los Angeles, California, United States of America; 5 Urologic Section, Department of Surgery, Durham VA Medical Center, Durham, North Carolina, United States of America; 6 Jonsson Comprehensive Cancer Center, UCLA, Los Angeles, California, United States of America; 7 Urology Section, Department of Surgery, Greater Los Angeles Veterans Affairs Healthcare System, Los Angeles, California, United States of America; 8 Department of Pathology and Laboratory Medicine, Cedars-Sinai Medical Center, Los Angeles, California, United States of America; 9 Department of Pathology, Greater Los Angeles Veterans Affairs Health System, Los Angeles, California, United States of America; University of Alabama at Birmingham, UNITED STATES

## Abstract

**Background:**

Benign human prostate tubule-initiating cells (TIC) and aggressive prostate cancer display common traits, including tolerance of low androgen levels, resistance to apoptosis, and microenvironment interactions that drive epithelial budding and outgrowth. TIC can be distinguished from epithelial and stromal cells that comprise prostate tissue via cell sorting based upon Epcam, CD44, and CD49f antigenic profiles. Fetal prostate epithelial cells (FC) possess a similar antigenic profile to adult TIC and are capable of inducing tubule formation. To identify the TIC niche in human prostate tissue, differential keratin (KRT) expression was evaluated.

**Results:**

Gene expression data generated from Affymetrix Gene Chip human U133 Plus 2.0 array of sorted adult and fetal epithelial cells revealed KRT13 to be significantly enriched in FC and TIC compared to basal cells (BC) and luminal cells (LC) (p<0.001). Enriched KRT13 expression was confirmed by RT-PCR and cytospin immunostaining. Immunohistochemical analysis of KRT13 expression revealed rare KRT13^+^ epithelia throughout prostatic ducts/acini in adult tissue specimens and differentiated tubules in 24-week recombinant grafts, In contrast, abundant KRT13 expression was observed in developing ducts/acini in fetal prostate and cord-like structures composing 8-week recombinant grafts. Immunostaining of a prostate tissue microarray revealed KRT13^+^ tumor foci in approximately 9% of cases, and this subset displayed significantly shorter time to recurrence (p = 0.031), metastases (p = 0.032), and decreased overall survival (p = 0.004). Diagnostic prostate needle biopsies (PNBX) from untreated patients with concurrent bone metastases (clinical stage M1) displayed KRT13^+^ tumor foci, as did bone metastatic foci.

**Conclusions:**

The expression profile of KRT13 in benign fetal and adult prostate tissue and in recombinant grafts, as well as the frequency of KRT13 expression in primary and metastatic prostate cancer indicates that it may be a marker of a stem/progenitor-like cell state that is co-opted in aggressive tumor cells. KRT13 is enriched in benign stem-like cells that display androgen-resistance, apoptosis-resistance, and branching morphogenesis properties. Collectively our data demonstrate that KRT13 expression is associated with poor prognosis at multiple stages of disease progression and may represent an important biomarker of adverse outcome in patients with prostate cancer.

## Introduction

Keratin (KRT) genes encode a diverse group of intermediate filament (IF) proteins that comprise the cytoskeleton of epithelial cells in order to maintain cellular structure in times of mechanical and non-mechanical cell stress [1, 2]. In addition to their well-characterized mechanical functions, newer studies suggest that KRTs may exhibit functional roles in apoptosis, cell growth, epithelial polarity, wound healing, and tissue remodeling [2, 3]. Over 50 unique KRTs are divided into two subgroups based upon the molecular weight and isoelectric point (pI) of the protein. Type I KRTs (9–19) are acidic and low molecular weight (LMW), ranging in size from 40 to 64 kDa. Type II KRTs (1–8) are high molecular weight (52–67 kDa) and are basic or neutral. Type I and II KRTs form heterodimer pairs within epithelial cells and often demonstrate both tissue-specific and cell type-restricted expression patterns, the regulation of which, is largely unknown[2]. KRTs are often utilized to determine cancer cell of origin or as prognostic indicators for certain malignancies, including breast, lung, and urothelial cell carcinoma, to name a few [3, 4].

The distribution of KRTs at various developmental stages may suggest cellular lineage as well as hierarchical epithelial differentiation. Prostate development begins following the outgrowth of the stem cell rich urogenital sinus, which stimulates outgrowth of small glandular buds [5]. These epithelial cord-like structures display a basal profile, marked, in part by predominant expression of KRT5, KRT6, and KRT14 [6, 7]. Branching morphogenesis and maturation of prostatic buds generates ducts and acini composed of distinct basal and luminal layers. In the mature adult prostate, epithelial cells that comprise the basal compartment express KRT5/KRT14 and reside adjacent to the basement membrane separating epithelia from fibromuscular stroma. Basal cells also contain a stem-like population, capable of inducing new, fully differentiated tubules [8, 9]. Luminal cells express KRT8/KRT18 and are terminally differentiated secretory cells located adjacent to the lumen of prostatic ducts/acini. In addition to basal and luminal cells, there exists an intermediate population of transit-amplifying cells, characterized by both basal and luminal KRT expression [7, 10]. In prostate cancer initiation, emergence of a discontinuous KRT5 (most prominent CK5) basal layer is a hallmark of the transformation process and defines precancerous prostate intraepithelial neoplasia (PIN) lesions[11]. When the basal cell layer is lost completely, epithelial outgrowths composed of abnormal luminal cell structures are deemed invasive adenocarcinoma. Accordingly, prostate cancer (PC) foci are generally characterized by expression of luminal keratins (KRT 8/18) and absence of KRT5/14[12].

In this report, KRT13, which is not described in prostate tissue, was found enriched in benign human prostate stem-like tubule-initiating cells (TIC). TIC are a subset of Epcam^+^CD49f^Hi^ basal cells that are characterized by absent CD44 expression and an enhanced ability to induce tubules in prostate recombination assays[9]. The observations of KRT13 expression in localized PC linked to poor outcome, primary PC tumor foci of metastatic cases, bone metastases, and prostate epithelial cells present in prostate glands following radiation and androgen deprivation therapy (ADT) suggest that KRT13 expression is associated with aggressive PC.

## Materials and Methods

### Prostate tissue procurement

Human subject research protocols and consent forms were approved by institutional review boards (IRBs) in the Office for the Protection of Research Subjects at UCLA and the Research and Development Committee at the Greater Los Angeles VA (GLA-VA) Healthcare System. Written informed consent was obtained from all participants in which surgical samples were prospectively procured. Archival tissue obtained in an anonymous fashion without identifiers did not require written or verbal consent, as determined, by the IRBs at UCLA and GLA-VA. Fetal prostate tissue samples (approximately 14–18 weeks gestation) were obtained in accordance with federal and state guidelines from the Center for AIDS Research at UCLA. Prostate tissue was preserved in frozen/paraffin blocks or dissociated to obtain single cell suspensions as previously described [6]. For benign adult prostate cell fractionation studies, surgical specimens were allocated for research by pathology and frozen sections of adjacent tissue were prepared in order to confirm areas of benign prostate glands. If cancer tissue was present, macrodissection and isolation of benign tissues was performed under the guidance of a genitourinary pathologist as previously described [13].

### Tissue processing

Mechanical and enzymatic digestion was performed on freshly isolated prostate tissue specimens, followed by sequential filtration and needle passage as previously described [8]. Resulting single cell suspensions were stored in RPMI supplemented with 10% FBS prior to cell sorting. Primary human fetal prostate cells (hFPS) cells were cultured in RPMI supplemented with 10% FBS and R1881 (Sigma, St Louis, MO, USA) and passaged three times prior to use in prostate tissue recombination assays.

### Cell fractionation and cytospin

Single cells were suspended in *PBS*, 2mM *EDTA*, *0*.*5*% *BSA* and stained with antibody for 15 minutes at 4°C. Fluorescence-activated cell sorting and analysis were performed on a BD Special Order FACS Aria II system and Diva v6.1.1 (BD Biosciences, San Jose, CA, USA). Live single cells were gated based on scatter properties and analyzed for their surface marker expression as previously described [9]. Cells were sorted and collected at 4°C using 100 μm nozzle and 23psi. Antibodies used for FACS include Epcam-PE (Miltenyi Biotech, San Diego, CA, USA), CD44-FITC (eBioscience, San Diego, CA, USA), and CD49f-APC (BioLegend, San Diego, CA, USA). Fractionated cells were used for cytospin and immunostaining or RNA collection (described below). Cell fractionation and subsequent analysis were performed on a total of 14 individual prostate tissue specimens, including 8 for the Affymetrix array analysis (described below), 3 for RT-PCR analysis (described below), and 3 for cytospin analysis. Cytospins were performed using a Shandon Cytospin 3 cytocentrifuge (Thermo Fisher, Canoga Park, CA, USA). Briefly, Shandon microscope slides, cytospin chambers, and blotters were prepared for each sample. Cell fractions (approximately 5x10^5^/mL) were suspended in 1X phosphor-buffered saline (PBS) and 200 microL was added to each slide chamber. Centrifugation at 800 rpm was performed, followed by air-drying of slides and immunostaining.

### Prostate tissue regeneration in immunocompromised mice

*In vivo* tissue regeneration experiments were performed in male SCID-NOD^IL2γrNULL^ mice in accordance with protocol number 2007-189-13, approved by the Animal Research Committee within the Office for the Protection of Research Subjects at UCLA. Mice (6–8 weeks old) were acquired from the UCLA Radiation Oncology Mouse Facility and housed in the Vivarium at UCLA. Animal care is under the supervision of the Attending Veterinarian. As detailed in the approved protocol, all SCID-NOD^IL2γrNULL^ mice received sterile food, water, bedding, and cage set-up. They were housed on hepa-filtered racks. Mice were anesthetized with isoflurane prior to undergoing subcutaneous injection of freshly isolated adult prostate cells and fetal prostate stroma cells suspended in Matrigel^®^ (Corning, Tewksbury, MA, USA) as previously described [8]. Euthanasia with isoflurane followed by cervical dislocation was performed between 6 and 24 weeks post-implantation. Recombinant grafts were isolated, formalin-fixed, and paraffin-embedded prior to sectioning for immunohistochemical analysis. All studies were performed in triplicate, using dissociated prostate tissue specimens from at least 3 independent patient specimens.

### RNA isolation, microarray hybridization, and data analysis

RNA was extracted from fractionated cells using Qiagen RNAeasy^®^ Micro Kit (Qiagen, Valencia, CA, USA), following the manufacturer’s instructions. After RNA extraction, all quantitation and microarray experiments were performed at the UCLA Department of Pathology Clinical Microarray Core Laboratory as previously described using Affymetrix Gene Chip U133Plus 2.0 Array (Affymetrix, Santa Clara, CA, USA) [6]. The Partek Genomics Suite Version 6.4 (Partek, St Louis, MO, USA) was employed and conducted using Partek default settings. RNA was extracted from FC, TIC, BC, and LC fractions as previously described[6]. A comparison of TIC versus BC and LC differentially expressed genes (n = 853) were selected at ≥ 2-fold difference. In order to evaluate the impact of FC, analysis of these 853 genes was performed using data from the pooled FC population (FC and TIC versus BC and LC). The list of the top 60 significantly enriched genes is shown in S1 Table. ANOVA analysis and t-test were used in order to identify differentially expressed genes with P<0.05.

### RT-PCR analysis

For quantitative Real-time PCR, RNA was isolated as described above. The concentration and purity of total RNA was assessed via UV spectrophotometer (260 and 280 nm). Total RNA (up to 5 μg) was used to generate cDNA via SuperScript III First-Strand Synthesis Kit (Thermo-Fisher, Waltham. MA, USA). For quantitative Real-time PCR, SYBR^®^-Green Supermix (Bio-Rad Laboratories, Hercules, CA, USA) was utilized with a Bio-Rad CFX Multicolor Real-time PCR detection system. PCR primer pairs for KRT13 were purchased from SABiosciences Corporation (Frederick, MD, USA). The PCR reaction conditions were performed as previously described [9].

### Immunohistochemistry

Prostate tissue was paraffin embedded as previously described [8]. Four-micron thick sections paraffin embedded tissue were deparaffinized with xylene and rehydrated through a descending series of ethanol washes as previously described. KRT13 staining was tested on positive control tissue (human tonsil) and negative control tissue (human colon) in order to confirm specificity of staining and optimize antibody dilution (data not shown). Western blot analysis with KRT13^+^ A431 cell control was performed as previously described and confirmed no cross-reactivity between KRT13 antibodies (Abcam, San Francisco, CA, USA, clones ab92551, 1x10^4^ dilution and ab133340, 1x10^4^ dilution) and other cytokeratins (S2 Fig)[14]. KRT5 (BioLegend, San Diego, CA, USA, clone PRB-160-P) and KRT8 (Abcam, San Francisco, CA, USA, clone ab53280) antibodies were used in immunohistochemical analysis at 1:200 and 1:2000 dilutions, respectively. Additional immunostaining performed: PSA (DAKO, Carpentaria, CA, USA, clone A0562, 1:200); P63 (Santa Cruz, CA, USA, clone 4A4, 1:200). Both manual and automated immunostaining was performed with equivalent results. Antigen retrieval and standard immunoperoxidase procedures were used, followed by detection with streptavidin peroxidase (Biogenex, Fremont, CA, USA) per manufacturer’s instructions.

### Tissue microarray (TMA) construction and analysis

Radical prostatectomy specimens obtained from 332 men undergoing surgery for prostate cancer between 1991–2003 at the GLA-VA were used for TMA construction, as previously described [15]. Briefly, 0.6-mm coring needles were used to collect areas of benign, prostatic intraepithelial neoplasia (PIN), and cancer from formalin-fixed paraffin-embedded (FFPE) surgical blocks. Between 4–15 cores were collected from each case with a total of approximately 3167 cores analyzed for KRT13 expression. Four-micron sections were cut from each TMA block for immunohistochemical staining, after optimization of KRT13 immunostaining on control tissues (described above). Stained slides were scanned using the Aerioslide scanner (Aperio, CA, USA). Evaluation of tumor foci was performed by two independent GU pathologists in a blinded fashion. Tumor foci present in cancer cores where >10% of cells expressed detectable KRT13 were annotated as KRT13 positive (+). HGPIN lesions were also evaluated and scored for KRT13 expression (1^+^ to 3^+^). A cancer core was deemed KRT13^+^ if tumor glands were observed in the core and any KRT13 staining was observed in tumor cells. HGPIN cores were designated KRT13^+^ if any of the HGPIN lesions demonstrated KRT13 staining. The intensity of the staining or the number of cells stained was not considered in the designation of KRT13^+^ or KRT13^-^ core. Clinical outcomes including biochemical recurrence, development of castration-resistant prostate cancer, development of clinical metastasis, and overall survival were retrieved from GLA-VA Medical Center databases as previously described. Biochemical recurrence was defined as a prostate specific antigen (PSA) value of 0.2 ng/mL, or a secondary treatment for an elevated post-operative PSA. 129/332 patients recurred and 14 developed metastases, with an average follow-up of 120 months.

### Additional statistical analysis

Data from the WLA cohort and TMA were used to determine relationships between KRT13 expression and clinical outcomes. Patients with positive KRT13 tumors were compared with patients who did not have positive KRT13 tumors across the following time to event outcomes: metastasis, overall survival, CRPC, and recurrence. Kaplan-Meier estimated probabilities for survival (metastasis-free, overall, CRPC-free, recurrence-free) at 1,3,5,and 6 years post-treatment were calculated between KRT13 positive and negative patients. We also constructed Kaplan-Meier curves and performed log-rank tests. Statistical analyses were carried out using IBM SPSS V23 (IBM Corp. Armonk, NY). P-values <0.05 were considered statistically significant.

## Results

### Gene expression analysis demonstrates specific KRTs are enriched in prostate epithelial subpopulations

**Fig 1:**
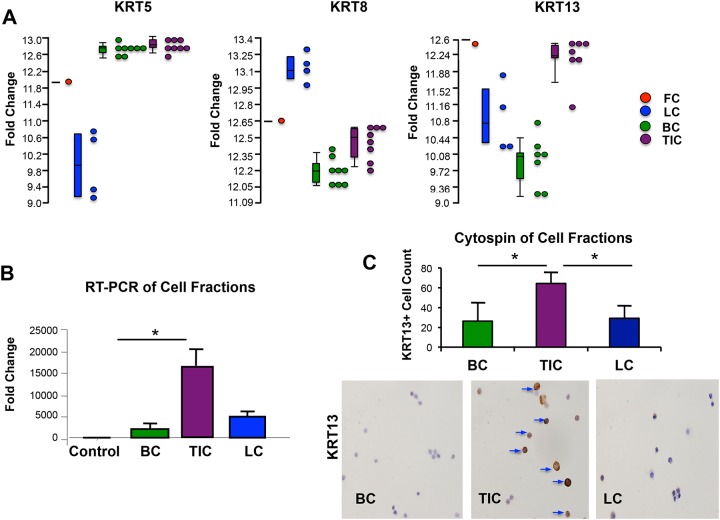
Differential KRT expression in fractionated prostate epithelial cells. (A). Differential expression of the basal KRT5, luminal KRT 8 and KRT 13 among the four cell populations analyzed (FC-red dot, LC-blue dots, BC-green dots, and TIC-purple dots) from the Affymetrix Gene Chip Human U133 PLUS 2.0 Array analysis. Each dot represents an individual sample, with the exception of the FC, where 6 samples were pooled to generate sufficient RNA for analysis. (B) RNA was isolated from sorted cell fractions generated from 3 unique prostate tissue specimens and evaluated via Quantitative RT-PCR. Unfractionated total prostate cell control is represented by the black column, Epcam^+^CD44^+^CD49f^Hi^ basal cells (BC) represented by green column, Epcam^+^CD44^-^CD49f^hi^ tubule initiating cells (TIC) represented by purple column, and Epcam^+^CD44^-^CD49f^Lo^ luminal cells (LC) represented by blue column. TIC had significantly higher expression than BC (p<0.05). (C) KRT13 immunostaining of cytospin slides of sorted cell fractions (BC, TIC, and LC) demonstrates relative abundance of KRT13^+^ cells, designated by brown staining, within the TIC fraction relative to BC and LC. The bar graph demonstrates the average cell count of KRT13^+^ cells per/cytospin slide (average of 3–5 slides, P<0.05).

We recently reported results from the gene expression profile analysis of functionally distinct epithelial subpopulations isolated from fetal and adult prostate specimens [6]. In the current report, we analyzed a list of 853 genes with ≥2-fold expression and p-value <0.05 that were differentially expressed in Epcam^+^CD44^-^CD49f^+^ fetal cells (FC) and adult TIC compared to Epcam^+^CD44^+^CD49f^Hi^ basal cells (BC) and Epcam^+^CD44^-^CD49f^Lo^ luminal cells (LC) to identify genes that may enable TIC to be identified in prostate tissue specimens (data not shown). The genes were reviewed with a focus on differentially expressed KRTs as potential markers of the TIC niche. As described above, KRT are useful in identifying specific compartments of the prostate, including KRT5 and KRT8, which are known to express in adult prostate basal and luminal cells, respectively[7, 8]. KRT13 demonstrated approximately 4.07-fold greater expression (p<0.001) in FC and TIC compared to BC and LC (S1 Table). A dotplot representing differences in expression of KRT13 in FC (pooled fractions, n = 6), as well as in each individual adult prostate cell fraction analyzed by microarray (7 TIC, 7 BC, and 4 LC) was compared to the expression of basal KRT5 and luminal KRT8 in order to highlight differences based upon antigenic profile (Fig 1A). As expected, both basal subpopulations (BC and TIC), as well as FC demonstrated enrichment of KRT5. In contrast, fractionated luminal cells (LC) expressed relatively low levels of KRT5, but demonstrated enrichment for KRT8 expression. KRT13 was enriched in TIC and FC but not in BC, which indicates an expression pattern that is distinct from KRT5 or KRT8. Quantitative RT-PCR demonstrated significantly increased expression of KRT13 in an independent set of fractionated cells isolated from benign prostate tissue from 3 surgical specimens (Fig 1B). Compared to total, unfractionated cell controls, KRT13 expression was significantly elevated in TIC relative to BC (P<0.05), but did not quite reach statistical significance in LC (p = 0.07). This result could reflect the challenge of obtaining pure separation of Epcam+CD44-CD49fHi from Epcam+CD44-CD49fLo cells with cell sorting. However, cytospins prepared from 3 pooled fractions collected from additional specimens and stained with KRT13 antibodies demonstrated significant enrichment of KRT13^+^ cells in the TIC fraction compared to both BC and LC (p<0.05, Fig 1C). Taken together, these results support KRT13 enrichment in the TIC population.

### KRT13 expression in prostate tissue and recombinant grafts identifies TIC niche

**Fig 2:**
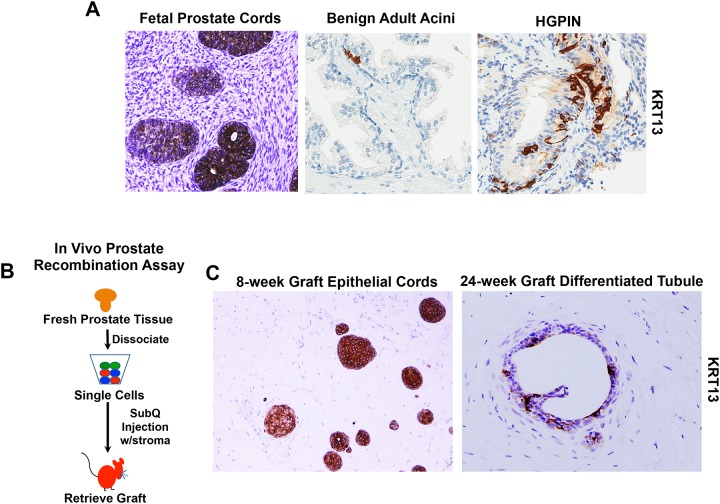
IHC analysis of KRT13 expression in fetal and adult prostate tissue and recombinant grafts. (A) Immunohistochemical (IHC) analysis of fetal prostate tissue, benign adult prostate tissue, and HGPIN lesion. Representative images of fetal tissue 14–18 weeks gestation are shown. KRT13+ Benign and HGPIN lesions were routinely observed in radical prostatectomy specimens. (B) Schematic of *in vivo* prostate recombination assay. All grafts were generated from freshly dissociated prostate cells combined with hFPS and injected subcutaneously with Matrigel^™^ *in vivo*. (C) IHC analysis of recombinant grafts stained for KRT13. 8-week graft demonstrates predominantly cord-like structures, and 24-week grafts demonstrate differentiated tubules with lumens. Grafts generated from 3 individual prostate specimens were collected at each time period (<12 weeks and >24 weeks). Representative images of KRT13 expression are shown.

Since KRTs are known for their ability to distinguish basal and luminal epithelial compartments, we investigated whether or not the TIC niche could be identified by KRT13 immunostaining in fetal and adult prostate tissue specimens (Fig 2A). In the fetal prostate, branching morphogenesis begins at approximately 11 weeks of gestation, and solid epithelial buds/cords are evident [5]. Exponential growth, triggered by increasing androgen levels, occurs throughout the second and third trimesters, suggesting that epithelial cells residing in fetal prostate tissue are poised to respond to hormonal cues and initiate branching morphogenesis. Consistent with the notion that the majority of fetal prostate cells possess tubule-initiating activity, similar to adult TIC, KRT13 was abundantly expressed throughout the epithelium of developing prostatic buds and cord-like structures (Fig 2A). In contrast, benign regions of adult prostate tissue, isolated from radical prostatectomy specimens, demonstrated scant, focal KRT13^+^ cells, which generally resided in the basal epithelial cell layer. Strong KRT13 expression was observed in high-grade prostatic intraepithelial neoplasia (HGPIN) lesions (Fig 2A and S1 Fig), although adjacent cancers rarely expressed KRT13, as described below.

### Recombinant prostate grafts demonstrate evolution of KRT13 expression in epithelial cords and differentiated tubules

Prostate tissue recombination assays are often utilized to identify cells with stem-like traits, capable of inducing tubules when combined with stroma and implanted subcutaneously into immunocompromised mice (schematic in Fig 2B) [16–18]. We have previously used these assays to identify TIC, a subpopulation of basal cells [6]. Recombinant grafts generated from freshly isolated prostate cells from benign surgical specimens that were combined with fetal prostate stroma and Matrigel were harvested 6–24 weeks following implantation (Fig 2C). Grafts harvested at 6–8 weeks post-implantation appeared to be predominantly composed of epithelial cord-like structures, containing underdeveloped or absent lumens. On the other hand, 24-week recombinant grafts demonstrated more differentiated ductal/acini. KRT13 immunostaining of these recombinant grafts demonstrated markedly different patterns of expression in epithelial cells that was reminiscent of the differences observed in fetal and adult prostate tissue (Fig 2A). That is, in less developed acini and cord-like structures, KRT13^+^ cells were predominant, similar to what was observed in fetal prostate tissue. In contrast, when tubules appeared more fully developed and contained both basal and luminal compartments (24-week grafts), KRT13^+^ cells are far less abundant, similar to the observations with benign adult prostate tissue.

### KRT13 expression is rare in localized prostate cancer, but commonly observed in prostate needle biopsies (PNBX) from cases associated with metastatic disease

**Fig 3:**
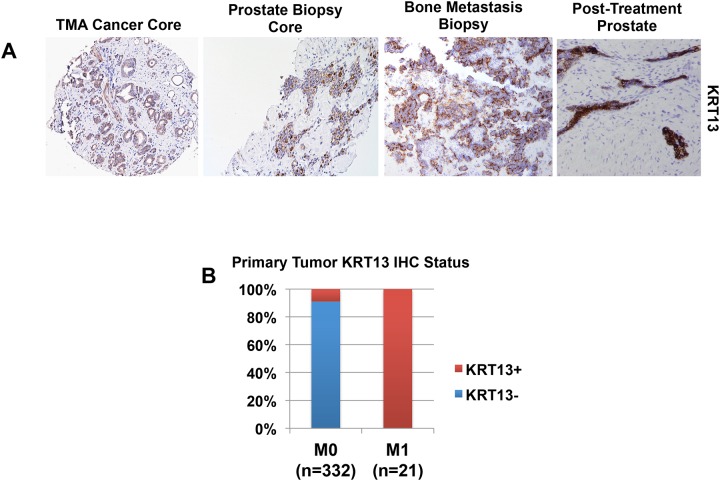
KRT13 expression in prostate cancer. (A) KRT13 expression is detected (from right to left) in a cancer core from the WLA TMA, a diagnostic PNBX core from a patient with diffuse bone metastases, a biopsy of a bone metastatic site, and residual prostate glands in a prostate specimen from a patient treated with radiation and ADT. (B) Bar graph representing the relative abundance of KRT13^+^ tumor foci identified in localized (stage M0) and metastatic (stage M1) cases. KRT13^+^ foci are identified in approximately 9% of prostate cancer cores from M0 cases in the WLA TMA. In contrast, all diagnostic PNBX from patients with diffuse bone metastases display KRT13^+^ tumor foci.

In order to determine if KRT13 is expressed in PC, tissue specimens from radical prostatectomy specimens, diagnostic PNBX of primary prostate tumors, and PC bone metastases were collected and subjected to immunostaining (Fig 3A). The Greater Los Angeles Veteran’s Affairs Healthcare System tissue microarray (WLA TMA) contains cancer cores from 332 men with localized prostate cancer who underwent radical prostatectomy [15]. Tumor cores from 199 cases are Gleason sum 8 or greater. The WLA TMA is linked to clinical outcome with over 12 years of follow-up. KRT13^+^ cancer foci were relatively rare, found in only 30/332 (approximately 9%) of cases (Fig 3). Interestingly, approximately 80% of PIN lesions were scored KRT13^+^ (data not shown). Some cases demonstrated both KRT13^+^ HGPIN and tumor foci, however, KRT13 expression was more often observed solely in HGPIN lesions (or benign glands), and while tumor foci were KRT13^-^ (S1 Fig). Men with metastatic prostate cancer at diagnosis (clinical stage M1) are generally not considered for surgical management. The standard of care treatment is initiation of androgen deprivation therapy (ADT). Consequently, the only source of cancer tissue that is available from these cases are diagnostic PNBX, and, rarely, biopsies of distant metastatic sites. KRT13 expression was evaluated in PNBX and bone metastasis biopsies obtained from M1 cases (Fig 3A). In contrast to the relatively low frequency of KRT13^+^ expression found in the WLA TMA cancer cores, PNBX cores from M1 cases displayed KRT13^+^ tumor foci in 21/21 cases (Fig 3B). Evaluation of tumors/HGPIN in prostate biopsies from patients with metastatic disease consistently showed KRT13^+^ HGPIN lesions adjacent to tumor foci (S1 Fig). Biopsies of untreated bone metastases from M1 patients (n = 3) also displayed KRT13 expression. Examination of a rare prostate tissue specimen retrieved at autopsy from a patient who died of prostate cancer, following treatment with radiation therapy and ADT revealed residual KRT13^+^ cells lining ducts/acini (Fig 3A). Taken together, these results indicate that KRT13 is prominent in tumor foci associated with aggressive disease. Interestingly, although KRT13 was discovered in TIC, which is a basal cell subpopulation, KRT13 expression in prostate tumors is associated with a luminal (PSA^+^ CK5^-^ P63^-^) phenotype (S2 Fig).

### Association between KRT13+ tumors and poor outcome in patients undergoing radical prostatectomy for localized prostate cancer

**Fig 4:**
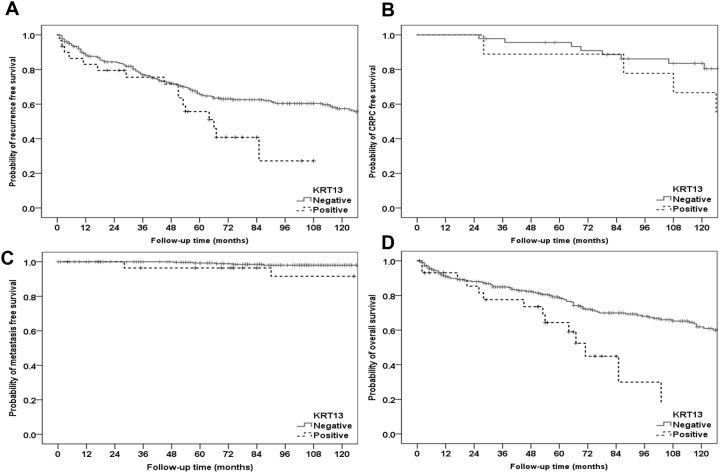
KRT13 expression in localized cancer is significantly associated with shorter time to recurrence. Kaplan Meier curves generated from outcomes of cases compiled in the WLA TMA and stratified based on the presence of KRT13^+^ tumor foci. A. Time to recurrence (P = 0.031). B. Time to CRPC (P = 0.331). C. Time to metastases (p = 0.032). D. Overall survival (p = 0.004).

Further analysis of clinical variables associated with the WLA TMA demonstrated significantly different outcomes with KRT13^+^ tumors. Kaplan Meier (KM) curves for recurrence, metastasis, CRPC, and overall survival are shown in Fig 4, while survival estimates for 1, 3, 5, and 6 years for each group are displayed in Table 1. The log-rank test was used to statistically compare the outcomes between KRT13^+^ and KRT13^-^ groups. Patients with KRT13^+^ tumors had shorter recurrence free survival (p = 0.031), increased metastatic progression (p = 0.032), and decreased overall survival (p = 0.004). Although there was a significant difference in CRPC progression using the chi-squared test (p = 0.050, data not shown), it was not significant using the log-rank test, which is being evaluated on a reduced sample size.

**Table 1:** Kaplan-Meier Estimated Probabilities for Survival.

Study Outcomes	KRT13	1-year survival	3-year survival	5-year survival	6-year survival	p-value
**Metastasis**	**-**	**1.00**	**1.00**	**0.99**	**0.99**	**0.032**
	**+**	**1.00**	**0.96**	**0.96**	**0.96**	
**Overall Survival**	**-**	**0.91**	**0.85**	**0.79**	**0.72**	**0.004**
	**+**	**0.93**	**0.77**	**0.64**	**0.46**	
CRPC*	**-**	**1.00**	**0.98**	**0.96**	**0.91**	**0.331**
	**+**	**1.00**	**0.89**	**0.89**	**0.89**	
**Recurrence**	**-**	**0.89**	**0.77**	**0.66**	**0.63**	**0.031**
	**+**	**0.83**	**0.76**	**0.55**	**0.39**	
*Reduced						

*Reduced Sample size

## Discussion

Prostate cancer associated with overt metastases at the time of diagnosis (clinical stage M1) or that becomes metastatic following localized treatment is lethal. Biomarkers indicative of lethal disease are needed to designate appropriate clinical management and develop new therapies for advanced disease. One approach to identifying potential biomarkers of aggressive prostate cancer is through identification and characterization of stem-like cells in benign prostate tissue that share features of aggressive prostate cancer and may serve as cells of origin or be co-opted in cancer. Numerous studies have demonstrated that prostate basal cells function as cells of origin of prostate cancer though genetic manipulation that induces expression of a variety of oncogenic combinations [13, 19–21]. Transduction of Trop2^+^CD49f^Hi^ prostate epithelial cells with activated AKT, ERG, and androgen receptor (AR) induces HGPIN and low-grade adenocarcinoma lesions in grafts obtained from prostate recombination assays [20]. Transduction of this same population with n-Myc induces high-grade lesions with neuroendocrine features [19]. The fact that basal cells are ripe for transformation and yield aggressive cancers suggests that they harbor traits that are advantageous to cancer cells.

Prostatic basal cells isolated from benign surgical specimens display low AR and do not require androgen for survival *in vitro* or *in vivo* [8]. They can form spheres in low attachment cultures or Matrigel and induce grafts composed of benign ducts/acini in recombination assays *in vivo* [8, 22]. Our previous studies have demonstrated that benign basal cells can be further divided into specific functional subpopulations via cell sorting with Epcam, CD44, and CD49f[9]. This fractionation approach enables separation of TIC (Epcam^+^CD44^-^CD49f^Hi^), which display superior ability to regenerate prostate grafts *in vivo* from BC (Epcam^+^CD44^+^CD49f^Hi^), which are more proliferative in sphere-forming assays *in vitro*.

TIC isolated from fetal and adult prostate tissue are enriched for stem-like features, including concomitant tolerance of low androgen levels and androgen responsiveness, resistance to apoptosis, and microenvironment interactions that drive epithelial budding and outgrowth [6, 8]. Our previous work demonstrated that gene expression analysis of fractionated adult and fetal prostate epithelial cells cluster together based upon antigenic profile. Importantly, FC displays a gene expression profile that more closely resembles adult TIC than BC and LC [6]. Similar to TIC, FC also display similar *in vivo* recombination function, in that they are capable of inducing tubular outgrowth. Taken together, these features of TIC suggest they are a subset of basal cells with a stem cell-like state. As such, features of TIC may be helpful in understanding the mechanisms used in aggressive cancer.

In the current study, we sought to examine differential KRT expression among these epithelial subpopulations in order to determine the location of functional cell types in prostate ducts/acini throughout development. We chose to focus on KRTs because they are commonly used to differentiate basal and luminal compartments of the prostate and designate areas of prostate cancer in pathology [23]. Upon examination of the list of genes with the most significant differential expression generated from a previous Affymetrix array analysis of fractionated prostate epithelial cells, KRT13 appeared to be significantly enriched in TIC. Subsequent analysis confirmed KRT13 enrichment in this subpopulation and revealed that its expression in localized prostate cancer, although relatively rare, is associated with poor outcome (recurrence, metastasis, and overall survival). Most striking is the finding that KRT13 is consistently detected in diagnostic biopsies of M1 patients, in addition to untreated bone metastases and residual prostate glands treated with ADT and radiation. These findings suggest that KRT13 may be a biomarker of aggressive disease.

One observation related to KRT13 expression in cancer specimens is the often discordant relationship between its expression in HGPIN and cancer, that is, KRT13 expression in HGPIN is significantly more prevalent than it is in tumor foci. The relationship between KRT13 and HGPIN may be important to consider in tumor initiation. It is possible that benign KRT13+ epithelial cells serve as cells of origin for prostate cancer. As discussed above, previous studies have demonstrated that the basal population is susceptible to malignant transformation[20]. Perhaps maintanence of KRT13 expression in tumors is dependent upon the degree of differentiation that occurs during tumor progression. If cancers become more differentiated and less aggressive, KRT13 expression should be lost in tumor cells. On the other hand, if tumor cells remain in a ‘stem-like’ state, characterized by KRT13 expression, they could be more prone to metastasis or resistance to ADT. Interestingly, KRT13^+^ cancers cells consistently display a luminal profile, with absent P63 expression and strong KRT8 and PSA. These results suggest that KRT13 may re-emerge in aggressive tumors. Future studies that evaluate the effect of direct transformation of benign KRT13^+^ prostate epithelial cells may reveal the importance of KRT13 expression in tumor evolution.

KRT13 is a 52 KDa type I keratin that often pairs with type II KRT4 [24, 25]. KRT13 is expressed in suprabasal layers of non-cornified stratified squamous epithelium, including mucosa lining the oral cavity, tonsils, larynx, esophagus, bladder, and lower female genital tract [25]. In cancer, KRT13 expression is found in well-differentiated urothelial carcinoma (poorly differentiated tumors were negative for KRT13), 10% of squamous carcinomas, and Brenner’s tumors [25]. KRT13 expression is regulated by calcium, nuclear receptor ligands, estradiol, and selective estrogen receptor modulators [24, 26]. KRT13 mutations are associated with the development of White Sponge Nevus, a condition characterized by thick white oral lesions [27]. Gradual loss of KRT13 expression in oral mucosa is linked to dysplastic transformation and squamous cell carcinoma [28]. Few cell lines exist that express *de novo* KRT13; however, the most well studied is the A431 epidermoid cancer cell line. A431 cells co-express high levels of epidermoid growth factor receptor (EGFR) and lack functional p53 [29]. Interestingly, intracardiac injection of these cells into immunocompromised mice results in rapid development of bone metastases, resulting in death within 3 weeks [30]. The mechanism by which bone metastases develop and the role of KRT13 in this process is unknown.

Keratins may play a functional role in cancer progression through cell signaling and skeletal organization. Aberrant expression of KRT8 and KRT18 is associated with neoplastic progression and invasion in squamous cell carcinomas [31]. Studies in breast cancer indicated that KRT19, one of the main cytoskeleton proteins of epithelial cells, is released by viable tumor cells, which may constitute a biologically active subset of breast cancer cells with high metastatic properties [32]. KRT8 is highly expressed in a multidrug-resistant breast cancer cell line, MCF-7/MX compared to parental MCF-7 cells, resulting in enhanced cell adhesion to the extracellular matrix, which could contribute to the mechanism of resistance [33, 34]. Keratin cytoskeletal organization mediated by casein kinase 1a (CK-1a) and a keratin-associated protein, FAM83H is aberrantly regulated in colon cancer, resulting in keratin filament disassembly and cancer progression [35].

Whether KRT13 is simply a biomarker of a stem-like state that is associated with aggressive disease or actually plays a functional role in metastatic progression of prostate cancer remains to be determined, as is further validation of KRT13 as a potential prognostic biomarker for aggressive prostate cancer. Specifically, a larger cohort of cases with clinical outcomes available could enable construction of a predictive model for metastatic progression that includes KRT13 expression status as a variable along with Gleason score and PSA levels. This report provides a foundation and rationale for future interrogation of KRT13 in prostate cancer progression.

## Supporting Information

S1 Fig KRT13 expression in HGPIN. Images of the cores from the WLA TMA demonstrate KRT13 immunostaining in HGPIN (brown staining). A. HGPIN lesion displays KRT13 staining in basal cells that extend toward the lumen. B. Prostate cancer core that is KRT13^-^ adjacent to KRT13^+^ HGPIN lesion. C. KRT13^+^ HGPIN lesion adjacent to KRT13^+^ and KRT13^-^ tumor foci. D. PNBX core from a patient with concurrent metastatic disease shows KRT13^+^ HGPIN adjacent to KRT13^+^ poorly differentiated tumor.
(TIF)

S2 Fig KRT13 antibody specificity. Antibodies are specific to KRT13 and demonstrate differences in expression in benign prostate tissues and cancer. A. Western blot analysis of protein collected from A431 cells, which are known to express KRT13 as well as multiple other cytokeratins was performed using two different KRT13 antibodies. A specific 50kDa band that is consistent with KRT13 protein (ladder is depicted in middle lane). B. KRT13 expression in fetal prostate tubules demonstrates majority of KRT13+ glands are also KRT5+, but KRT8-. C. KRT13 expression correlates with basal profile in adult benign prostate, with staining in basal cells that are P63+ and KRT8-, PSA- (top panel). In contrast, KRT13 expression in cancer demonstrates a luminal profile, with co-expression of KRT8 and PSA, but lack of P63 staining (bottom panel).
(TIF)

S1 Table Top 60 enriched genes from 853 gene set TIC and FC versus LC and BC. This table indicates the highest fold differences in gene expression identified in the Affymetrix microarray analysis of fractionated prostate epithelial cells (TIC and FC versus BC and LC). ANOVA analysis and t-test were used in order to identify 853 significantly differentially expressed genes with (P<0.05). Keratin 13 (KRT13) is included in the top 60 with 4.06-fold increased expression (p<0001).
(XLSX)
