# Supplementary figures and images for: Keratin 13 Is Enriched in Prostate Tubule-Initiating Cells and May Identify Primary Prostate Tumors that Metastasize to the Bone
Source: PLoS One. 2016 Oct 6;11(10):e0163232. doi: 10.1371/journal.pone.0163232 (PMC5053503; doi:10.1371/journal.pone.0163232)

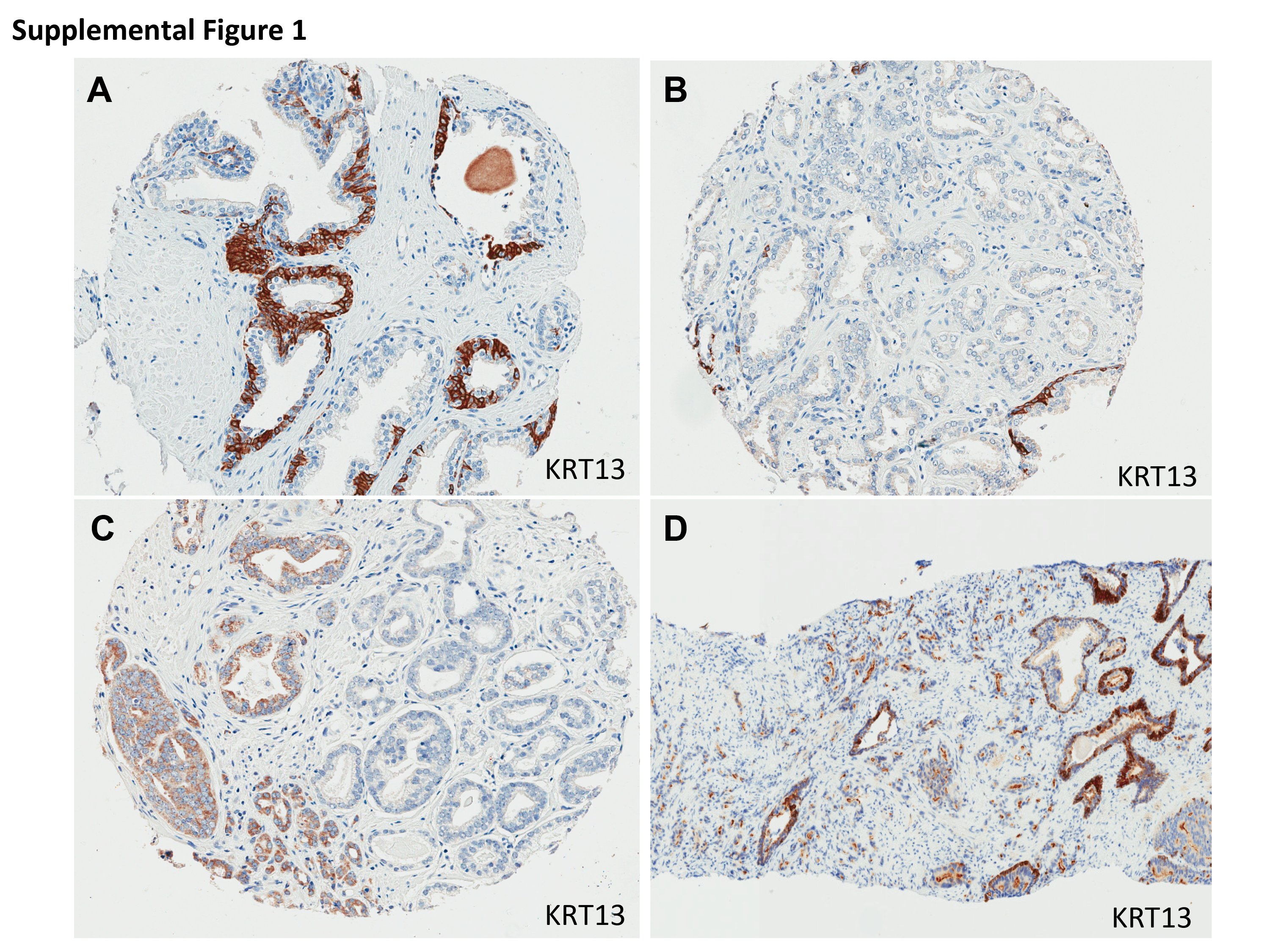

Supplement: S1 Fig — Images of the cores from the WLA TMA demonstrate KRT13 immunostaining in HGPIN (brown staining). A. HGPIN lesion displays KRT13 staining in basal cells that extend toward the lumen. B. Prostate cancer core that is KRT13- adjacent to KRT13+ HGPIN lesion. C. KRT13+ HGPIN lesion adjacent to KRT13+ and KRT13- tumor foci. D. PNBX core from a patient with concurrent metastatic disease shows KRT13+ HGPIN adjacent to KRT13+ poorly differentiated tumor. (TIF) [file pone.0163232.s001.tif]

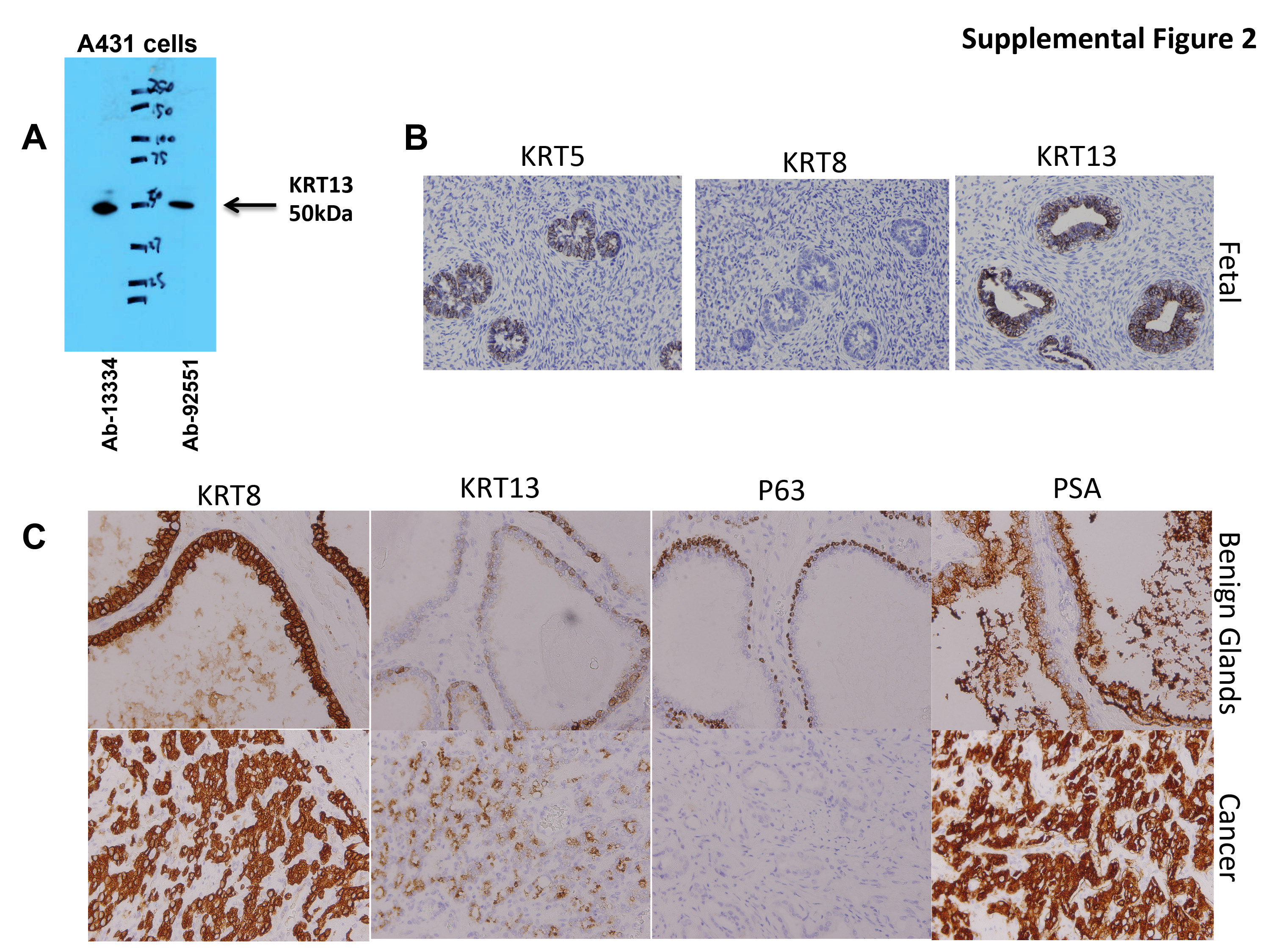

Supplement: S2 Fig — Antibodies are specific to KRT13 and demonstrate differences in expression in benign prostate tissues and cancer. A. Western blot analysis of protein collected from A431 cells, which are known to express KRT13 as well as multiple other cytokeratins was performed using two different KRT13 antibodies. A specific 50kDa band that is consistent with KRT13 protein (ladder is depicted in middle lane). B. KRT13 expression in fetal prostate tubules demonstrates majority of KRT13+ glands are also KRT5+, but KRT8-. C. KRT13 expression correlates with basal profile in adult benign prostate, with staining in basal cells that are P63+ and KRT8-, PSA- (top panel). In contrast, KRT13 expression in cancer demonstrates a luminal profile, with co-expression of KRT8 and PSA, but lack of P63 staining (bottom panel). (TIF) [file pone.0163232.s002.tif]
